# The Long-Term Relationship between Population Growth and Vegetation Cover: An Empirical Analysis Based on the Panel Data of 21 Cities in Guangdong Province, China

**DOI:** 10.3390/ijerph10020660

**Published:** 2013-02-07

**Authors:** Chao Li, Yaoqiu Kuang, Ningsheng Huang, Chao Zhang

**Affiliations:** 1 Sustainable Development Research Center, Guangzhou Institute of Geochemistry, Chinese Academy of Sciences, Guangzhou, Guangdong 510640, China; E-Mails: lcyw41020118@163.com (C.L.); nshuang@gzb.ac.cn (N.H.); 2 Key Laboratory of Marginal Sea Geology, Chinese Academy of Sciences, Guangzhou, Guangdong 510640, China; 3 University of Chinese Academy of Sciences, Beijing 100049, China; 4 Guangdong Social Sciences Association, Guangzhou, Guangdong 510050, China; E-Mail: kaikudan@126.com

**Keywords:** population growth, vegetation cover, inverted N-shaped curve, NDVI

## Abstract

It is generally believed that there is an inverse relationship between population growth and vegetation cover. However, reports about vegetation protection and reforestation around the World have been continuously increasing in recent decades, which seems to indicate that this relationship may not be true. In this paper, we have taken 21 cities in Guangdong Province, China as the study area to test the long-term relationship between population growth and vegetation cover, using an AVHRR NDVI data set and the panel cointegrated regression method. The results show that there is a long-term inverted N-shaped curve relationship between population growth and vegetation cover in the region where there are frequent human activities and the influence of climate change on vegetation cover changes is relatively small. The two turning points of the inverted N-shaped curve for the case of Guangdong Province correspond to 2,200 persons·km^−2^ and 3,820 persons·km^−2^, and they can provide a reference range for similar regions of the World. It also states that the population urbanization may have a negative impact on the vegetation cover at the early stage, but have a positive impact at the later stage. In addition, the Panel Error Correction Model (PECM) is used to investigate the causality direction between population growth and vegetation cover. The results show that not only will the consuming destruction effect and planting construction effect induced by the population growth have a great impact on vegetation cover changes, but vegetation cover changes in turn will also affect the population growth in the long term.

## 1. Introduction

Vegetation plays an important role in energy conversion and material circulation of the Earth, and vegetation cover is a visual sign of ecosystem health [1]. Although the dynamic evolution of vegetation cover is affected by both natural and anthropogenic factors [2,3,4], the impact of anthropogenic factors on the vegetation cover change is more significant than that of natural factors in the regions with frequent human activities [5,6,7,8].

The available studies about population and vegetation cover have mainly focused on the destructive effects of human activities on vegetation [9,10,11,12]. Green vegetation was excessively logged and used by human beings to support their productive and living activities [13]. Industrial growth in urban areas, including highway and railway construction, water use, mining and power generation, have directly consumed a huge amount of vegetation and caused chemical pollution [14,15]. The development of agriculture has destroyed a large part of the original vegetation, such as forests [16,17]. Tourism activities do not only lead to the death of some plants, but also subsequently exert a great negative effect on the growth of plants [18,19,20]. The destructive effects of human activities on vegetation have exerted an increasingly severe impact on the ecological environment, accordingly resulting in grassland degradation, deforestation, desertification, farmland reduction, soil erosion and other serious consequences [21].

During recent years, studies concerning how human activities were improving vegetation cover began to increase with more vegetation protection and reforestation [22,23,24,25]. Population growth does not necessarily mean vegetation destruction, because economic, social, political, technological and other developments can actually promote reforestation and improve vegetation cover [26,27,28]. A study has suggested that the forest covers of Europe, North America and Asia were increasing, and more and more countries and regions were shifting from deforestation to reforestation [29]. Some data from France indicated that although the population had been growing, there had been an upward trend in forest cover due to ongoing reforestation activities in France since 1830 [30]. From 1960 to 2006, the French forest area expanded more than a quarter, while the total population increased from 42 million to 61 million [29].

In summary, human activities can either increase or reduce the vegetation cover, which in the long term may therefore not just show an inverse relationship between population growth and vegetation cover as many scholars have stated [31,32,33,34], and a U-shaped or inverted N-shaped curve relationship might be more possible, yet, empirical research on this issue are quite few. Some studies have already investigated a positive relationship between population growth and vegetation cover in recent decades [35,36], but they all used statistical forest cover indexes which may cause inaccurate and non-comprehensive results. Firstly, the forest cover cannot reflect the cover of other vegetation types in an area, such as arable lands, grasslands and gardens. Secondly, the forest cover also cannot reflect the vegetation cover of urban areas with frequent human activities. Thirdly, the diverse statistical standards of forest cover index and other human factors will result in errors. NDVI is an indicator of vegetation’s growth state and spatial distribution, and has a positive correlation with vegetation cover. It reflects all the vegetation cover on land, including forests, arable lands, grasslands, gardens and parks. A greater NDVI value indicates higher vegetation cover and better vegetation growth [37,38]. Therefore, this paper uses NDVI instead of forest cover indexes to reflect vegetation cover and takes 21 cities in Guangdong Province, China as the study area to test the long-term relationship between population growth and vegetation cover, using the panel cointegrated regression method. 

## 2. Materials and Methods

### 2.1. Study Area

**Figure 1:**
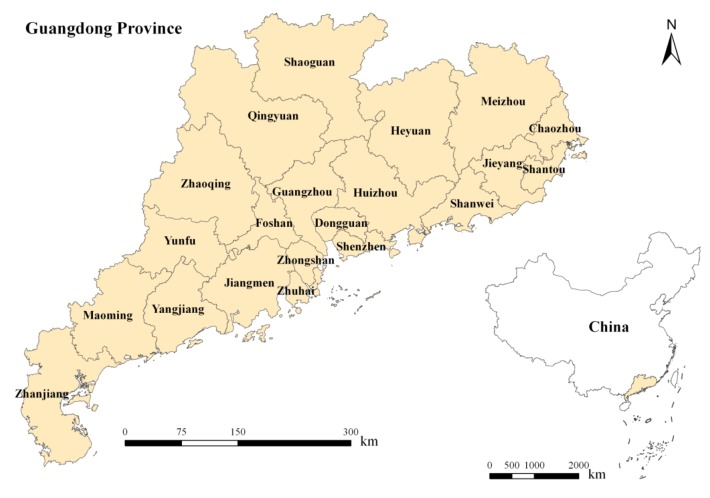
Map showing the geographic location of the study area.

The studied area covers the entire Guangdong Province in China (20°13′N–25°31′N, 109°39′E–117°19′E) (Figure 1). The province, with a land area of 179,757 km^2^, is located in southeast China and ranked fifteenth in size among all the Chinese provinces. It is divided into 21 administrative units called cities, and each city is used as the basic unit to analyze the vegetation cover and population characteristics.

The surface of the province is dominated by forests, accounting for 58.9% in 2006. Meanwhile, arable lands, grasslands, urban and built-up lands, and bodies of water account for 22.4%, 0.2%, 9.8% and 4.7%, respectively [39].

The climate of the province is of a humid subtropical monsoon type [40,41]. Its annual precipitation is generally over 1,300 mm and annual average temperature is about 22 °C. Due to the adequate rainfall, mild weather and forest-dominated landscape, the influence of climate change on the vegetation cover changes in this region is small [42].

As the forefront area of China’s reform and opening up, Guangdong Province has maintained rapid population growth since the 1980s, with annually average population growth rate above 2.27%. Due to the diversity of economic development and geographical environment, there is a significant difference in population growth among 21 cities of the province. The population grew rapidly in the developed Pearl River Delta Region, and, for example, Shenzhen’s permanent residents grew from 0.45 million to 8.46 million in 1982–2006, increasing by 1.783% in just 25 years; the population grew steadily in western Guangdong, and Zhanjiang’s permanent residents have grown from 4.49 million to 6.90 million in 1982–2006, increasing by 53.7% in 25 years; finally, the population grew slowly in northern Guangdong, and Shaoguan’s permanent residents have grown from 2.43 million to 2.95 million in 1982–2006, increasing by 21.2% in 25 years. This population growth diversity among the 21 cities made Guangdong Province form different population distribution patterns at different periods. This phenomenon provides us with an ideal panel data set to carry out empirical research on the long-term relationship between population growth and vegetation cover.

### 2.2. Data Sources and Processing

NDVI is a ratio of the near-infrared (NIR) and visible (VIS) radiances. It is calculated as: NDVI = (NIR − VIS)/(NIR + VIS). The NDVI data set used in this study was provided by the Environmental and Ecological Science Data Center for West China, National Natural Science Foundation of China (http://westdc.westgis.ac.cn). It is generated from the Global Inventory Monitoring and Modeling Studies (GIMMS) group, derived from the NOAA/AVHRR land data set, at a spatial resolution of 8 km × 8 km and taken at half a month intervals, for the period from January 1982 to December 2006. This data set has been processed with radiometric correction, geometric correction, replacing bad lines and cloud clearing. In order to explore inter-annual variations in vegetation cover, we use the Maximum Value Compositing (MVC) technique to reconstruct the half-monthly NDVI data set into the annual maximum NDVI data set. Data of population density is calculated by city according to the Guangdong Statistical Yearbooks from 1982 to 2006 (Table A1). The annual maximum NDVI values were averaged on the surface of the 21 cities to get yearly time series by cities (Table A2). 

**Figure 2:**
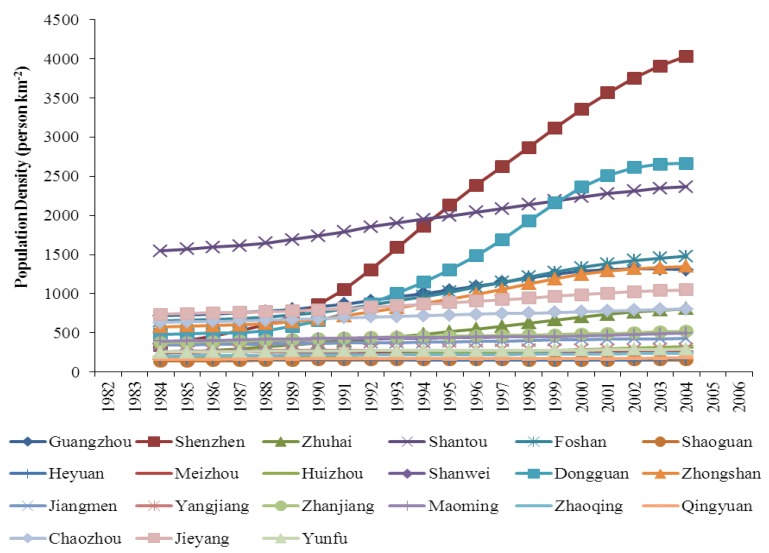
Trends in population density of 21 cities during the period from 1982 to 2006. Note: A 5-year smoothing interval is used to clearly show the trends.

**Figure 3:**
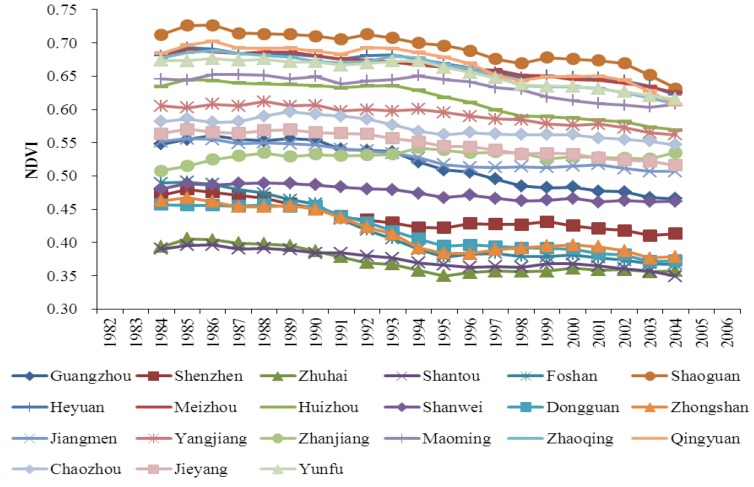
Trends in NDVI of 21 cities during the period from 1982 to 2006. Note: A 5-year smoothing interval is used to clearly show the trends.

The trends in population density and NDVI among the 21 cities in the study area are diverse during the period from 1982 to 2006 (Figure 2, Figure 3). Some cities with fast population growth have experienced different NDVI trends from the cities with slow population growth. This phenomenon provides us an ideal panel data set to carry out the empirical research on the long-term relationship between population growth and vegetation cover. The statistical characteristics of the panel data are shown in Table 1.

**Table 1:** Descriptive statistics of population density and annual maximum NDVI data of 21cities in Guangdong Province during the period from 1982 to 2006.

	*P* (Population Density)	*V* (NDVI)
Mean	0.661006	0.588819
Median	0.425	0.62016
Maximum	4.287	0.75056
Minimum	0.134	0.350488
Std. Dev.	0.673703	0.097254
Observations	525	525
Cross sections	21	21

### 2.3. Methods

Based on a conceptual model, we constructed a simplified cubic polynomial regression equation to test the long-term relationship between population growth and vegetation cover, using the panel cointegrated regression method. In order to ensure the effectiveness of the fitting model, the two most commonly used panel unit root test methods, including the LLC test [43] and Fisher-ADF test [44], are applied to test the stationarity of the panel data (the LLC test is applicable for homogeneous panels, while the Fisher-ADF test is applicable for heterogeneous panels). Three kinds of panel cointegrated test methods, including Pedroni [45], Kao [46] and Johansen-Fisher [47], are applied to determine whether there is a long-term cointegrated relationship between population growth and vegetation cover or not. The random effects regression model is used to estimate the regression equation. The Panel Error Correction Model (PECM) is used to investigate the causality direction between population growth and vegetation cover.

## 3. Conceptual Model

The influences of population growth (the population growth here includes the natural population growth and immigration growth) on vegetation cover can be considered as two effects. One is the consuming destruction effect. Population growth can inevitably result in increasing demands for life necessities. To meet these demands, large areas with good vegetation cover will be exploited for construction of houses, roads, factories and shops, and many vegetation resources will be plundered, resulting in a vegetation cover decrease [5]. Another is the planting construction effect. Vegetation is an essential element for human development, and it can help improve the living environment as well as providing productive materials and a source of energy for humans. With the population growth, the demands for the ecological functions provided by vegetation will increase. To fulfill these demands, some constructive actions, such as vegetation protection, reforestation and so on, will be carried out, resulting in a vegetation cover increase [48].

Of course, vegetation cover can be influenced by other factors, especially the long-term inter-annual climate changes, which affect vegetation cover mainly through fluctuation of rainfall. However, in the studied area, the annual rainfall was more than 1,300 mm during the period from January 1982 to December 2006. It is considered where there are frequent human activities and the influence of climate change is relatively small on vegetation cover changes (anthropogenic factors play a dominant role in the vegetation cover changes in this region, while the natural factors have a small impact). Based on the above-stated two effects, we try to construct a conceptual model as follows: the long-term relationship between population growth and vegetation cover can be separated into three stages in a region where there are frequent human activities and the influence of climate change on vegetation cover changes is small. At the first stage, there is an inverse relationship between population growth and vegetation cover. As the vegetation cover is relatively high and the public facilities are imperfect at the early stage of population growth, the vegetation cover decreases fast with the population growth when the consuming destruction effect is much stronger than the planting construction effect. Subsequently, due to the improvement of public facilities, the consuming destruction effect induced by population growth tends to become small. Besides, the ecological deterioration resulting from vegetation cover decrease makes people aware of the importance of the ecological functions of vegetation. To improve the vegetation cover, the government implements some measures, such as more intensive residence areas, vegetation protection, reforestation and so on. Consequently, the planting construction effect gradually offsets the consuming destruction effect, and the inverse relationship between population growth and vegetation cover weakens at the latter part of the first stage. 

At the second stage, there is a positive relationship between population growth and vegetation cover. With the development of society and the economy, the ecological functions of vegetation arouse more people’s attention. Then, some new green technologies are developed, which makes many places without vegetation cover (such as roofs, walls, fences, bridges, *etc.*) be planted and the permanent vegetation cover protected. At the same time, as the population continues to grow and the government strengthens the vegetation protection, to meet the need for vegetation resources, the food and resource sectors tend to increase imports, resulting in a further decline of the consuming destruction effect. These activities cause an increase of vegetation cover. At this stage, the planting construction effect directly or indirectly induced by population growth has already exceeded the consuming destruction effect, while the relationship between population growth and vegetation cover becomes positive, and the vegetation cover tends to increase slightly with the population growth.

At the third stage, there is an inverse relationship between population growth and vegetation cover again. With the population continuing to expand beyond a certain limit, the consuming destruction effect will again surpass the planting construction effect, and then vegetation cover will tend to decrease with population growth.

**Figure 4:**
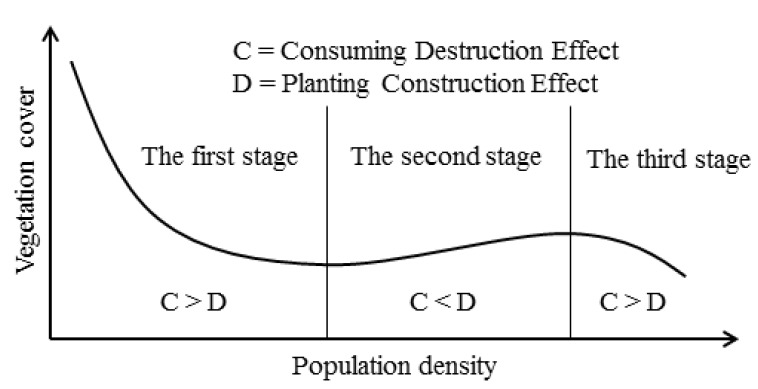
The long-term inverted N-shaped curve relationship between population growth and vegetation cover.

In the long term, we will observe an inverted N-shaped curve relationship between population growth and vegetation cover (Figure 4). What needs to be explained is that, with the economic development and birth rate changes, the third stage may not appear in some regions because of the zero or negative population growth. In these regions, the vegetation cover may be maintained at the level of the second stage or even improved with the population decrease.

To test the long-term inverted N-shaped curve relationship between population growth and vegetation cover, we constructed a simplified cubic polynomial regression equation after the Environmental Kuznets Curve (EKC) [49] as follows:

V = c + β_1_P + β_2_ P^2^ + β_3_ P^3^ + u (1)

where *V* (NDVI) is the index reflecting the vegetation cover, *P* (population density) is the index reflecting the population, c is a constant, u is the random error, the parameters *β_1_*, *β_2 _*and *β_3_* are the coefficients of the first, second and third term of *P,* respectively. If *β_3_* > 0, *β_2_* < 0, *β_1_* > 0, there is a N-shaped curve relationship between population growth and vegetation cover, which means that the vegetation cover tends to increase first, then decrease and rise again with the population growth. If *β_3_* < 0, *β_2_* > 0, *β_1_* < 0, there is an inverted N-shaped curve relationship between population growth and vegetation cover, which means that the vegetation cover tends to decrease first, then increase and decline again with the population growth. If *β_3_* = 0, *β_2_* > 0, *β_1_* < 0, there is an U-shaped curve relationship between population growth and vegetation cover, which means that vegetation cover tends to decrease first and then increase with the population growth. If *β_3_* = 0, *β_2_* < 0, *β_1_* > 0, there is an inverted U-shaped curve relationship between population growth and vegetation cover, which means that the vegetation cover tends to increase first and then decrease with the population growth. If *β_3_ = β_2_* = 0, *β_1_*≠ 0, there is a linear relationship between population growth and vegetation cover.

## 4. Results and Discussion

### 4.1. Cointegration Relationship

In order to ensure the effectiveness of the fitting model, we test the stationarity of the panel data. The results (Table 2) show that all variables in the panel series are unable to reject the null hypotheses of non-stationarity at the specified significance levels, while all the 1st differential panel series reject the null hypotheses of non-stationarity at a significance level of 10% at least. This indicates that all variables can be considered as non-stationary panel series with first order integration (denoted as I(1)).

**Table 2:** Test statistics of the panel unit root test for the variables involved in Equation (1).

Test method	Levin, Lin & Chu t		ADF - Fisher Chi-square	
Panel series	Primary	1st difference	Primary	1st difference
*V*	1.04913	−30.3697 ***	36.4358	595.897 ***
*P*	−2.89544 ***	−3.74377 ***	27.5211	61.1483 **
*P* ^2^	−0.52219	−3.02647 ***	23.647	56.5126 *
*P* ^3^	1.56007	−2.35949 ***	23.0499	57.9320 *

The primary panel series are tested with intercept, while the 1st differential panel series are tested without intercept; *, ** and *** indicate that the null hypotheses of a unit-root are rejected at significance level of 10%, 5% and 1% respectively.

Since all variables are non-stationary panel series with first order integration (I(1)), we proceed with the panel cointegration test to determine whether there is a long-term relationship between population density and NDVI or not. The results (Table 3) show that all tests reject the null hypotheses of no cointegration among the four variables at significance level of 5%, which suggests that there is a long-term cointegration relationship between NDVI and population density.

**Table 3:** Panel cointegration tests.

	Panel				Group		
	v-St	rho-St	PP-St	ADF-St	rho-St	PP-St	ADF-St
Pedroni	−2.677	−3.7234 ***	−10.0978 ***	−3.9372 ***	−2.9945 ***	−17.3462 ***	−7.0680 ***
Kao	t-Statistic: 1.7008 **						
Fisher	Fisher Stat. *: 474.2 ***						

** and *** indicate that the null hypotheses of no cointegration are rejected at 5% and 1% levels respectively.

### 4.2. A Long-term Inverted N-shaped Curve

**Figure 5:**
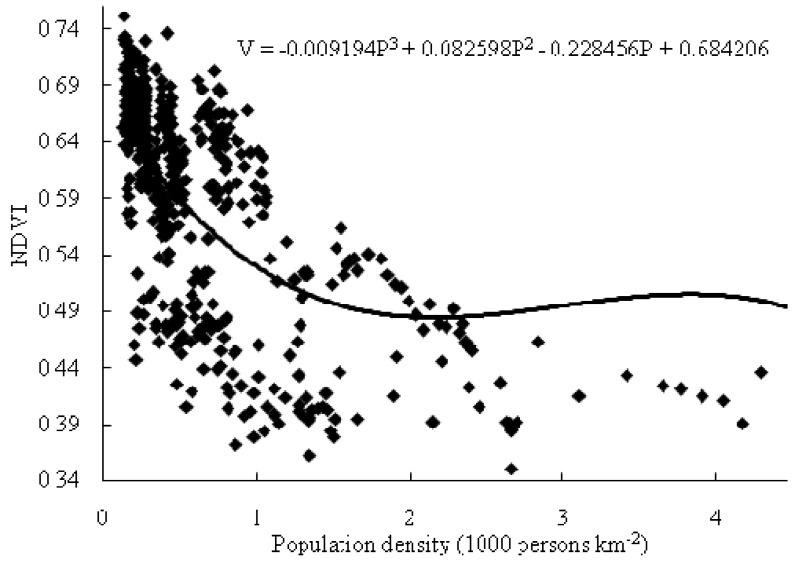
The panel regression curve of population density and NDVI.

We use the Hausman test to determine whether a fixed or random effects model is appropriate to estimate the Equation (1), and the result shows that the null hypothesis of a random effects model isn’t rejected at 10% significance level. So we use the random effects model to estimate the equation, and the regression results (Table 4) show that there is a long-term inverted N-shaped curve relationship between population growth and vegetation cover, because the null hypotheses that the coefficients of variables are equal to zero are all rejected at a significance level of 1%, and the coefficients meet the condition *β_3_* < 0, *β_2_* > 0 and *β_1_* < 0. The panel regression curve of population density and NDVI is shown in Figure 5. The first turning point, where the relationship between population density and NDVI changes from negative to positive correlation, occurs at the population density of about 2,200 persons·km^−2^. The second turning point, where the relationship between population density and NDVI changes from positive to negative correlation, occurs at the population density of about 3,820 persons·km^−2^. Most of the 21 cities were still at the first stage when there was an inverse relationship between population growth and vegetation cover. However, as the government continued to strengthen environmental and vegetation protection, some cities with a higher population density, such as Guangzhou, Zhuhai, Foshan, Zhongshan and so on, had entered the latter part of the first stage when the devegetation process had slowed down. Dongguan and Shantou had entered the second stage when the relationship between population growth and vegetation cover became positive. Shenzhen, whose population density was 4,287 persons·km^−2^ and vegetation cover was higher than that of Dongguan and Shantou, had just entered the third stage when the inverse relationship between population growth and vegetation cover appeared again.

**Table 4:** Estimation results from the panel random effects regression.

Variable	*P*	*P* ^2^	*P* ^3^	*c*
Coefficient	−0.228456 ***	0.082598 ***	−0.009194 ***	0.684206 ***
	(−8.819210)	−5.592608	(−3.940781)	−41.59666
R-squared	0.334048			
F-statistic	87.11297 ***			

*** indicates that the null hypothesis that the coefficient is zero is rejected at 1% level.

In contrast to other studies’ inverse relationship [31,32,33,34], our empirical result shows that there is a long-term inverted N-shaped curve relationship between population growth and vegetation cover. It should be noted that 2,200 persons·km^−2^ and 3,820 persons·km^−2^ are the two turning points of the inverted N-shaped curve based on the experiences of Guangdong Province’s 21 cities, where there are similar natural conditions and social systems. However, they can just provide a reference range for similar regions of the World, where there are also frequent human activities and the influence of climate change on vegetation cover changes is also small, because different social systems and natural conditions will affect the location of the two turning points in different regions. For example, there will be faster vegetation restoration and the turning point will come earlier in more democratic regions, where people’s ecological needs can be better reflected.

### 4.3. Relationships in Different Regions

**Figure 6:**
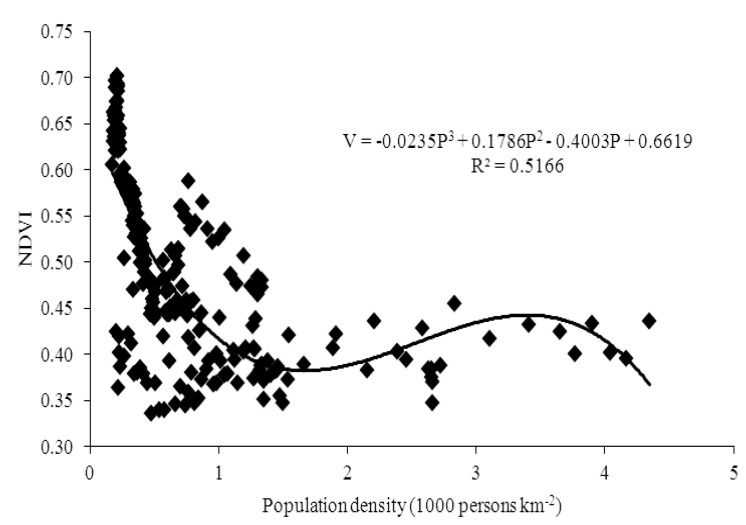
The scatterplot of population density and NDVI with fitted curve in high-urbanization cities from 1982 to 2006.

**Figure 7:**
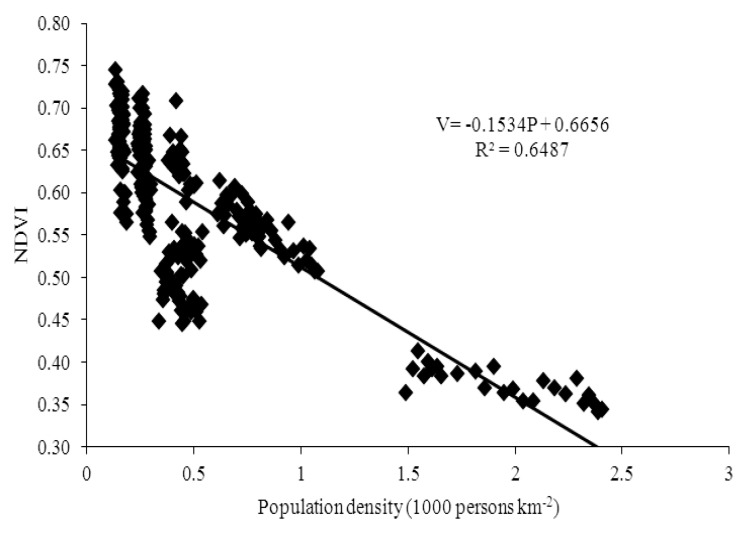
The scatterplot of population density and NDVI with fitted curve in low-urbanization cities from 1982 to 2006.

Guangdong is a province with unbalanced regional development, consisting of the developed Pearl River Delta region which has experienced a fast urbanization and the undeveloped peripheral region which has experienced a relatively slow urbanization. As there is big difference among 21 cities in different regions, we classify the cities into two types. One type is dominated by high-urbanization cities located in the Pearl River Delta region, including Guangzhou, Shenzhen, Zhuhai, Foshan, Huizhou, Dongguan, Zhongshan, Jiangmen and Zhaoqing. The other type is dominated by low-urbanization cities located in the peripheral region, including Shantou, Shaoguan, Heyuan, Meizhou, Shanwei, Yangjiang, Zhanjiang, Maoming, Qingyuan, Chaozhou, Jieyang and Yunfu. Then we draw the scatterplots of population density and NDVI with fitted curve, and find that there is an inverted N-shaped curve relationship between population growth and vegetation cover in the high-urbanization cities from 1982 to 2006 (Figure 6), while there is a negative linear relationship in the low-urbanization cities (Figure 7). Due to the free migration of population among cities in Guangdong province, the cities with higher urbanization would have larger population. Therefore, according to the conceptual model, we can explain that the cities with higher urbanization would enter the second and the third stage earlier. It also states that the population urbanization may have a negative impact on the vegetation cover at the early stage [50,51,52], but have a positive impact at the later stage.

### 4.4. Granger Causality

According to the Granger Representation Theorem [53], if the variables are cointegrated, there must be at least one direction of causal relationship between them. The results (Table 5) show that the null hypotheses that the coefficients of panel error correction terms *ecm*(*v*) and *ecm*(*p*) are equal to zero are rejected at significance levels of 1% and 5%, respectively, which indicates that *V* is Granger causal for *P* and *P* is Granger causal for *V* in the long term. 

**Table 5:** The estimated results of the Panel Error Correction Model (PECM).

D(V)		D(P)	
Variable	Coefficient	Variable	Coefficient
c	−0.005207 *** (−3.950969)	c	0.005245 *** (5.450232)
ecm(v)	−0.118505 *** (−2.715071)	ecm(p)	−0.005538 ** (−2.152945)
D[V(−1)]	−0.392603 *** (−6.946931)	D[V(−1)]	−0.038606 (−1.123531)
D[V(−2)]	−0.182085 *** (−3.956092)	D[V(−2)]	−0.000284 (−0.009368)
D[P(−1)]	−0.006660 (−0.111667)	D[P(−1)]	1.165810 *** (26.33352)
D[P(−2)]	−0.028675 (0.486901)	D[P(−2)]	−0.383236 *** (−8.155351)

*ecm*(*v*) and *ecm*(*p*) are the panel error correction terms; *ecm*(*v*) is the lag-1 panel series of residuals in fixed effects model regression of *V* on *P*, and *ecm*(*v*) is the lag-1 panel series of residuals in random effects model regression of *V* on *P*; We select the lag length in 2;* D*[V(−1)] and *D*[*V*(−2)] are the lag-1 and lag-2 1st differential panel series of *V* respectively; *D*[*P*(-1)]and *D*[*P*(−2)]are the lag-1 and lag-2 1st differential panel series of *P* respectively; ** and *** indicate that the null hypotheses that the coefficients of variables are equal to zero are rejected at 5% and 10% significance levels respectively.

We conclude that there is a long-term interactive relationship between population growth and vegetation cover. In other words, the impact of population growth on the vegetation cover change is a long-term process, and the long-term changes of vegetation cover will restrict the population growth. The null hypotheses that the regression coefficients of *D*(*V*) on *D*[*P*(−1)] and *D*[*P*(−2)], and *D*(*P*) on *D*[*V*(−1)] and *D*[*V*(−2)] are equal to zero are not rejected at significance level of 10% respectively, which indicates that *V* is not Granger causal for *P* and *P* is not Granger causal for *V* in the short term. We conclude that there is not a short-term interactive relationship between population growth and vegetation cover. In other words, the short-term changes of population may affect the vegetation cover changes little, while the short-term changes of vegetation cover may also affect the population little.

## 5. Conclusions

The purpose of this paper was to prove that there is a long-term inverted N-shaped curve relationship between population growth and vegetation cover in a region where there are frequent human activities and the influence of climate change is relatively small on vegetation cover changes [42]. In summary, we have obtained the following conclusions:

(1) There can be a positive relationship between population growth and vegetation cover. According to the empirical results of 21 cities in Guangdong Province, China, the vegetation cover will increase slightly with the population growth when the population density is in the range of 2,200–3,820 persons·km^−2^. Although the two turning points of population density from Guangdong Province’s experiences may not be entirely applicable to other parts of the World, it can provide a reference range for similar regions. At present, the global population is still growing, but some regions’ vegetation cover has been improved. In these regions, a lot of new technology (such as roof greening) is being developed to increase the vegetation cover, and many new measures (such as increasing imports) are implemented to reduce the consumption of vegetation resources. With the economic and social development, people are asking for greener environments and healthier living. As long as the population does not expand beyond a certain limit, the vegetation cover can remain unchanged or even improved in many regions. 

(2) Unlimited population growth will eventually lead to a significant reduction of vegetation cover. According to the empirical results of 21 cities in Guangdong Province, China, the vegetation cover will decrease with the population growth when the population density is below 2,200 persons·km^−2^ or above 3,820 persons·km^−2^. For some regions in the World, the population growths are still at the first stage and are leading to a significant reduction of vegetation cover on land. For other regions, although the vegetation covers are experiencing a recovery with more people paying close attention to the ecological functions of vegetation, the degrees of vegetation restoration are low. If the population growth continues to expand beyond a certain limit, the consuming destruction effect will surpass the planting construction effect again, and the inverse relationship between population growth and vegetation cover will appear eventually.

(3) Population urbanization may free up space for vegetation ecological construction at some stage. According to the empirical results, there is an inverted N-shaped curve relationship between population growth and vegetation cover in the high-urbanization cities while there is only a negative linear relationship in the low-urbanization cities. This result shows that the cities with higher urbanization may have chances to improve their vegetation cover at the later stage, when the land saving from the urban agglomeration effect is larger than the land occupation from the urban expansionary effect, and more people promote more green building.

(4) Population growth and vegetation cover affect each other in the long term. According to the results of the Granger causality test, there is an interactive relationship between population growth and vegetation cover in the long term, but not in the short term. This result shows that not only will the consuming destruction effect and planting construction effect induced by the population growth have a great impact on vegetation cover changes in the long term, but vegetation cover changes in turn will also affect the population growth. For example, the suburban areas with better vegetation cover and ecological environment are attracting more urban population to move into them.

Finally, this paper has just tested a long-term inverted N-shaped curve relationships between population growth and vegetation cover through the panel data of 21 cities in Guangdong Province, China, where there are frequent human activities and the climate is of a humid subtropical monsoon type. However, maybe there will be the same relationships in most of the regions where there are frequent human activities, including humid, sub-humid, semiarid and arid climate regions, because anthropogenic factors may also play a dominant role in the vegetation cover changes in these regions. Therefore, further study is necessary to provide validation using data from more regions.

## Appendix

**Table A1:** The average annual permanent population density of 21 cities in Guangdong Province from 1982 to 2006 (person·km^−2^).

Year	GZ	SZ	ZH	ST	FS	SG	HY	MZ	HZ	SW	DG	ZS	JM	YJ	ZJ	MM	ZQ	QY	CZ	JY	YF
1982	692	209	222	1,492	630	134	155	239	177	339	468	569	339	253	363	383	197	156	614	714	247
1983	703	267	226	1,522	638	135	158	244	180	347	474	573	341	256	369	388	200	159	623	727	250
1984	714	342	231	1,549	647	137	160	247	183	356	480	579	344	259	374	393	202	161	632	738	253
1985	727	415	239	1,574	656	140	162	250	186	364	486	585	347	262	380	397	205	163	640	748	256
1986	740	465	268	1,596	667	142	164	253	188	372	495	593	349	265	387	403	207	165	646	755	258
1987	754	510	302	1,616	679	144	166	256	191	380	503	601	353	268	394	410	210	167	653	762	262
1988	768	578	322	1,636	692	146	165	261	194	389	510	611	357	273	402	419	212	169	660	770	265
1989	782	670	344	1,657	704	148	164	265	197	398	518	621	361	277	409	428	215	171	668	779	268
1990	821	792	368	1,732	749	152	162	262	203	410	617	660	364	279	426	433	216	172	681	791	271
1991	873	1,010	393	1,819	809	156	160	257	211	420	762	717	368	279	441	434	216	172	694	808	272
1992	913	1,267	419	1,861	857	155	158	255	218	424	868	764	373	279	445	436	217	172	702	827	273
1993	955	1,546	448	1,904	907	154	156	253	225	429	988	814	377	279	449	439	218	171	710	845	273
1994	999	1,917	478	1,949	960	153	154	251	233	434	1,125	868	382	278	454	441	219	170	718	864	274
1995	1,045	2,207	510	1,994	1,016	153	153	249	240	438	1,282	925	387	278	458	443	221	169	727	884	274
1996	1,093	2,386	545	2,040	1,075	152	151	247	249	443	1,460	986	392	278	462	446	222	168	735	904	275
1997	1,143	2,588	582	2,088	1,148	151	149	245	257	448	1,662	1,051	396	278	467	448	223	167	744	924	275
1998	1,196	2,837	622	2,136	1,214	151	147	244	265	453	1,893	1,120	401	278	471	451	224	166	753	945	276
1999	1,251	3,105	664	2,186	1,274	150	146	242	274	458	2,156	1,194	406	278	476	453	225	166	761	967	276
2000	1,308	3,415	709	2,239	1,349	149	144	240	284	463	2,456	1,273	412	278	481	456	227	165	771	989	277
2001	1,339	3,651	747	2,291	1,407	150	147	242	294	475	2,635	1,319	418	281	491	465	230	168	783	1,013	281
2002	1,333	3,767	770	2,325	1,437	152	154	247	304	489	2,656	1,329	423	286	504	477	235	173	794	1,030	287
2003	1,317	3,904	789	2,345	1,456	154	160	251	312	501	2,657	1,339	425	289	513	486	239	177	799	1,041	291
2004	1,304	4,043	811	2,371	1,480	156	166	255	321	514	2,659	1,346	428	293	524	497	243	182	807	1,054	295
2005	1,288	4,170	831	2,391	1,501	158	173	258	329	526	2,661	1,350	430	296	533	506	246	186	812	1,065	299
2006	1,295	4,287	849	2,402	1,515	159	176	259	335	532	2,700	1,369	430	298	537	514	248	188	815	1,072	301

Data of average annual permanent population density are calculated according to Guangdong Statistical Yearbook from1981 to 2006; GZ, SZ, ZH, ST, FO, SG, HY, MZ, HZ, SW, DG, ZS, JM, YJ, ZJ, MM, ZQ, QY, CZ, JY and YF are short for Guangzhou, Shenzhen, Zhuhai, Shantou, Foshan, Shaoguan, Heyuan, Meizhou, Huizhou, Shanwei, Dongguan, Zhongshan, Jiangmen, Yangjiang, Zhanjiang, Maoming, Zhaoqing, Qingyuan, Chaozhou, Jieyang and Yunfu respectively.

**Table A2:** The annual maximum NDVI of 21 cities in Guangdong Province from 1982 to 2006.

Year	GZ	SZ	ZH	ST	FS	SG	HY	MZ	HZ	SW	DG	ZS	JM	YJ	ZJ	MM	ZQ	QY	CZ	JY	YF
1982	0.514	0.424	0.364	0.364	0.451	0.661	0.650	0.658	0.605	0.448	0.443	0.419	0.539	0.609	0.485	0.638	0.663	0.641	0.574	0.546	0.671
1983	0.560	0.504	0.419	0.392	0.513	0.728	0.713	0.711	0.662	0.507	0.476	0.502	0.579	0.623	0.516	0.667	0.689	0.687	0.614	0.598	0.699
1984	0.560	0.470	0.402	0.413	0.488	0.745	0.686	0.679	0.642	0.473	0.450	0.480	0.564	0.607	0.506	0.642	0.697	0.721	0.587	0.569	0.673
1985	0.557	0.476	0.386	0.383	0.489	0.726	0.676	0.668	0.631	0.480	0.460	0.446	0.527	0.576	0.502	0.633	0.667	0.695	0.560	0.552	0.652
1986	0.549	0.483	0.398	0.400	0.507	0.702	0.680	0.679	0.635	0.494	0.456	0.467	0.550	0.612	0.530	0.648	0.674	0.680	0.573	0.554	0.672
1987	0.548	0.467	0.422	0.392	0.458	0.730	0.710	0.710	0.653	0.489	0.439	0.443	0.560	0.596	0.522	0.630	0.702	0.701	0.597	0.579	0.669
1988	0.588	0.482	0.411	0.395	0.497	0.731	0.700	0.699	0.658	0.497	0.475	0.471	0.573	0.650	0.565	0.708	0.702	0.715	0.585	0.576	0.716
1989	0.536	0.445	0.379	0.383	0.450	0.684	0.654	0.665	0.621	0.485	0.444	0.443	0.536	0.593	0.533	0.642	0.675	0.671	0.594	0.560	0.660
1990	0.543	0.457	0.380	0.386	0.459	0.721	0.673	0.681	0.627	0.483	0.470	0.446	0.531	0.607	0.524	0.628	0.654	0.692	0.600	0.575	0.663
1991	0.565	0.440	0.385	0.389	0.458	0.700	0.680	0.676	0.630	0.490	0.442	0.473	0.543	0.580	0.503	0.620	0.659	0.681	0.607	0.558	0.649
1992	0.536	0.430	0.379	0.369	0.426	0.716	0.695	0.683	0.643	0.477	0.444	0.418	0.552	0.603	0.537	0.651	0.673	0.680	0.578	0.559	0.668
1993	0.522	0.421	0.369	0.394	0.384	0.706	0.677	0.675	0.641	0.481	0.400	0.407	0.542	0.604	0.554	0.651	0.690	0.691	0.571	0.568	0.693
1994	0.526	0.422	0.335	0.363	0.367	0.722	0.682	0.656	0.637	0.472	0.394	0.372	0.522	0.604	0.538	0.666	0.685	0.719	0.571	0.555	0.674
1995	0.534	0.436	0.368	0.368	0.393	0.694	0.675	0.664	0.629	0.478	0.405	0.393	0.512	0.598	0.531	0.636	0.693	0.686	0.557	0.544	0.680
1996	0.486	0.403	0.339	0.353	0.379	0.660	0.647	0.656	0.593	0.461	0.386	0.369	0.510	0.592	0.552	0.648	0.647	0.651	0.559	0.532	0.650
1997	0.476	0.428	0.339	0.353	0.368	0.697	0.666	0.668	0.593	0.445	0.389	0.378	0.499	0.578	0.520	0.625	0.621	0.646	0.551	0.524	0.618
1998	0.507	0.455	0.392	0.377	0.406	0.669	0.644	0.655	0.602	0.501	0.407	0.404	0.525	0.577	0.532	0.633	0.659	0.645	0.589	0.565	0.655
1999	0.473	0.417	0.346	0.369	0.373	0.658	0.647	0.654	0.579	0.448	0.383	0.403	0.519	0.583	0.543	0.622	0.629	0.628	0.559	0.531	0.634
2000	0.484	0.432	0.365	0.362	0.371	0.662	0.632	0.624	0.583	0.458	0.394	0.406	0.518	0.592	0.525	0.622	0.623	0.647	0.552	0.514	0.631
2001	0.472	0.424	0.344	0.380	0.377	0.704	0.665	0.650	0.589	0.465	0.384	0.382	0.503	0.562	0.509	0.589	0.639	0.678	0.560	0.537	0.636
2002	0.480	0.400	0.359	0.351	0.381	0.685	0.653	0.643	0.582	0.459	0.384	0.387	0.510	0.567	0.538	0.602	0.622	0.650	0.549	0.519	0.617
2003	0.478	0.433	0.380	0.361	0.383	0.660	0.641	0.649	0.586	0.475	0.375	0.389	0.535	0.585	0.526	0.610	0.644	0.648	0.566	0.534	0.638
2004	0.468	0.402	0.347	0.351	0.355	0.634	0.625	0.630	0.568	0.459	0.370	0.375	0.489	0.555	0.537	0.608	0.597	0.598	0.548	0.514	0.610
2005	0.438	0.395	0.350	0.341	0.347	0.576	0.589	0.600	0.544	0.448	0.347	0.351	0.498	0.554	0.520	0.608	0.586	0.564	0.537	0.507	0.603
2006	0.465	0.436	0.352	0.344	0.372	0.602	0.600	0.605	0.566	0.468	0.387	0.392	0.501	0.548	0.554	0.611	0.589	0.574	0.533	0.507	0.610

The data set is provided by Environmental and Ecological Science Data Center for West China, National Natural Science Foundation of China (http://westdc.westgis.ac.cn); GZ, SZ, ZH, ST, FO, SG, HY, MZ, HZ, SW, DG, ZS, JM, YJ, ZJ, MM, ZQ, QY, CZ, JY and YF are short for Guangzhou, Shenzhen, Zhuhai, Shantou, Foshan, Shaoguan, Heyuan, Meizhou, Huizhou, Shanwei, Dongguan, Zhongshan, Jiangmen, Yangjiang, Zhanjiang, Maoming, Zhaoqing, Qingyuan, Chaozhou, Jieyang and Yunfu respectively.

## References

[1] Cihlar J., Stlaurent L., Dyer J.A. (1991). Relation between the normalized difference vegetation index and ecological variables. Rem. Sens. Environ..

[2] Bradshaw R., Hannon G. (1992). Climatic-change, human influence and disturbance regime in the control of vegetation dynamics within Fiby Forest, Sweden. J. Ecol..

[3] Vicente-Serrano S.M., Lasanta T., Romo A. (2004). Analysis of spatial and temporal evolution of vegetation cover in the Spanish Central Pyrenees: Role of human management. Environ. Manag..

[4] Kulakowski D., Bebi P., Rixen C. (2011). The interacting effects of land use change, climate change and suppression of natural disturbances on landscape forest structure in the Swiss Alps. Oikos.

[5] Meyer W.B., Turner B.L. (1992). Human-population growth and global land-use cover change. Ann. Rev. Ecol. Syst..

[6] Vanderknaap W.O., Vanleeuwen J.F.N. (1995). Holocene vegetation succession and degradation as responses to climatic-change and human activity in the Serra de Estrela, Portugal. Rev. Palaeobot. Palyno..

[7] Wang Y.B., Feng Q., Si J.H., Su Y.H., Chang Z.Q., Xi H.Y. (2011). The changes of vegetation cover in Ejina Oasis based on water resources redistribution in Heihe River. Environ. Earth Sci..

[8] Peng J., Xu Y.Q., Cai Y.L., Xiao H.L. (2011). Climatic and anthropogenic drivers of land use/cover change in fragile karst areas of southwest China since the early 1970s: A case study on the Maotiaohe watershed. Environ. Earth Sci..

[9] Foster D.R., Zebryk T., Schoonmaker P., Lezberg A. (1992). Post-settlement history of human land-use and vegetation dynamics of a *Tsuga canadensis* (hemlock) woodlot in central new England. J. Ecol..

[10] Andersen U.V. (1995). Resistance of danish coastal vegetation types to human trampling. Biol. Conserv..

[11] Kissling M., Hegetschweiler K.T., Rusterholz H.P., Baur B. (2009). Short-term and long-term effects of human trampling on above-ground vegetation, soil density, soil organic matter and soil microbial processes in suburban beech forests. Appl. Soil. Ecol..

[12] Shipigina E., Rees W.G. (2012). Analysis of human impact on boreal vegetation around Monchegorsk, Kola Peninsula, using automated remote sensing technique. Polar Rec..

[13] Watson A. (1985). Soil-erosion and vegetation damage near ski lifts at cairn-gorm, Scotland. Biol. Conserv..

[14] Ratcliffe D.A. (1984). Post-medieval and recent changes in British vegetation—The culmination of human influence. New Phytol..

[15] Sasaki N., Takahara H. (2011). Late holocene human impact on the vegetation around Mizorogaike Pond in northern Kyoto basin, Japan: A comparison of pollen and charcoal records with archaeological and historical data. J. Archaeol. Sci..

[16] Delcourt H.R. (1987). The impact of prehistoric agriculture and land occupation on natural vegetation. Trends Ecol. Evol..

[17] Mann D., James E., Chase J., Beck W., Reanier R., Mass M., Finney B., Loret J. (2008). Drought, vegetation change, and human history on Apa Nui (Isla de Pascua, easter Island). Quat. Res..

[18] Burden R.F., Randerson P.F. (1972). Quantitative studies of the effects of human trampling on vegetation as an aid to the management of semi-natural areas. J. Appl. Ecol..

[19] Kerbiriou C., Leviol I., Jiguet F., Julliard R. (2008). The impact of human frequentation on coastal vegetation in a biosphere reserve. J. Environ. Manag..

[20] Lucas-Borja M.E., Bastida F., Moreno J.L., Nicolas C., Andres M., Lopez F.R., del Cerro A. (2011). The effects of human trampling on the microbiological properties of soil and vegetation in Mediterranean mountain areas. Land Degrad. Dev..

[21] Bai Z., Dent D. (2009). Recent land degradation and improvement in China. AMBIO.

[22] Rudel T.K. (1998). Is there a forest transition? Deforestation, reforestation, and development. Rural Soc..

[23] Nagendra H. (2007). Drivers of Reforestation in Human-Dominated Forests. Proc. Natl. Acad. Sci. USA.

[24] Cortina J., Amat B., Castillo V., Fuentes D., Maestre F.T., Padilla F.M., Rojo L. (2011). The restoration of vegetation cover in the semi-arid Iberian southeast. J. Arid. Environ..

[25] Plieninger T., Schleyer C., Mantel M., Hostert P. (2012). Is there a forest transition outside forests? Trajectories of farm trees and effects on ecosystem services in an agricultural landscape in eastern Germany. Land Use Pol..

[26] Mather A.S., Needle C.L. (1998). The forest transition: A theoretical basis. Area.

[27] Mather A.S., Fairbairn J. (2000). From floods to reforestation: The forest transition in Switzerland. Environ. Hist..

[28] Nagendra H., Nagendra H., Southworth J. (2010). Reforestation and regrowth in the human dominated landscapes of South Asia. Reforesting Landscapes: Linking Pattern and Process.

[29] Kauppi P.E., Ausubel J.H., Fang J., Mather A.S., Sedjo R.A., Waggoner P.E. (2006). Returning Forests Analyzed with the Forest Identity. Proc. Natl. Acad. Sci. USA.

[30] Mather A.S., Fairbairn J., Needle C.L. (1999). The course and drivers of the forest transition: The case of France. J. Rural Stud..

[31] Allen J.C., Barnes D.F. (1985). The causes of deforestation in developing countries. Ann. Ass. Am. Geogr..

[32] Harrison P. (1993). The Third Revolution: Environment, Population and a Sustainable World.

[33] DeFries R.S., Rudel T., Uriarte M., Hansen M. (2010). Deforestation driven by urban population growth and agricultural trade in the twenty-first century. Nat. Geosci..

[34] Hazra S., Sen R.K., Deb B.J. (2010). Population Growth and Forest: Degradation in North East India. Population and Development in North East India.

[35] Mather A., Needle C. (2000). The relationships of population and forest trends. Geogra. J..

[36] Nagendra H. (2010). Reforestation and regrowth in the human dominated landscapes of South Asia. Reforest. Landsc..

[37] Nordberg M.L., Evertson J. (2003). Monitoring change in mountainous dry-heath vegetation at a regional scale using multitemporal Landsat^TM^ data. AMBIO.

[38] Purevdorj T., Tateishi R., Ishiyama T., Honda Y. (1998). Relationships between percent vegetation cover and vegetation indices. Int. J. Rem. Sens..

[39] Land-Use Change Survey Statistics of Guangdong Province. http://www.gdlr.gov.cn/newsAction.do?method=viewNews&classId=020019980000000488&newsId=020010040000014133.

[40] Zheng J., Yin Y., Li B. (2010). A new scheme for climate regionalization in China. Acta Meteorolog. Sinic..

[41] Zhao S.-Q. (1983). A new scheme for comprehensive physical regionalization in China. Acta Geogr. Sinic..

[42] Li X.-B., Shi P.-J. (2000). Sensitivity analysis of variation in NDVI, temperature and precipitation in typical vegetation types across China. Acta Phytoecolog. Sinic..

[43] Levin A., Lin C.F., Chu C.S.J. (2002). Unit root tests in panel data: Asymptotic and finite-sample properties. J. Econometrics.

[44] Maddala G.S., Wu S.W. (1999). A comparative study of unit root tests with panel data and a new simple test. Oxford B Econ. Stat..

[45] Pedroni P. (2004). Panel cointegration: Asymptotic and finite sample properties of pooled time series tests with an application to the PPP hypothesis. Economet. Theor..

[46] Kao C. (1999). Spurious regression and residual-based tests for cointegration in panel data. J. Econometrics.

[47] Larsson R., Lyhagen J., Löthgren M. (2001). Likelihood-based cointegration tests in heterogeneous panels. Econometrics J..

[48] Fuller R.A., Gaston K.J. (2009). The scaling of green space coverage in European cities. Biol. Lett..

[49] Grossman G.M., Krueger A.B. Environmental Impacts of a North American Free Trade Agreement. http://www.nber.org/papers/w3914.

[50] Mohan M., Pathan S.K., Narendrareddy K., Kandya A., Pandey S. (2011). Dynamics of urbanization and its impact on land-use/land-cover: A case study of megacity delhi. J. Environ. Protec..

[51] Piao S., Fang J., Zhou L., Guo Q., Henderson M., Ji W., Li Y., Tao S. (2003). Interannual variations of monthly and seasonal normalized difference vegetation index (NDVI) in China from 1982 to 1999. J. Geophys. Res..

[52] Dewan A.M., Yamaguchi Y. (2009). Land use and land cover change in Greater Dhaka, Bangladesh: Using remote sensing to promote sustainable urbanization. Appl. Geogr..

[53] Granger C.W.J. (1988). Ome recent development in a concept of causality. J. Econometrics.

